# Inflorescence development in tomato: gene functions within a zigzag model

**DOI:** 10.3389/fpls.2014.00121

**Published:** 2014-03-31

**Authors:** Claire Périlleux, Guillaume Lobet, Pierre Tocquin

**Affiliations:** Laboratory of Plant Physiology, PhytoSYSTEMS, Department of Life Sciences, University of LiègeLiège, Belgium

**Keywords:** *Solanum lycopersicum*, flowering, morphogenesis, sympodial growth, biological model, AGL24

## Abstract

Tomato is a major crop plant and several mutants have been selected for breeding but also for isolating important genes that regulate flowering and sympodial growth. Besides, current research in developmental biology aims at revealing mechanisms that account for diversity in inflorescence architectures. We therefore found timely to review the current knowledge of the genetic control of flowering in tomato and to integrate the emerging network into modeling attempts. We developed a kinetic model of the tomato inflorescence development where each meristem was represented by its “vegetativeness” (V), reflecting its maturation state toward flower initiation. The model followed simple rules: maturation proceeded continuously at the same rate in every meristem (dV); floral transition and floral commitment occurred at threshold levels of *V*; lateral meristems were initiated with a gain of *V* (Δ*V*) relative to the *V* level of the meristem from which they derived. This last rule created a link between successive meristems and gave to the model its zigzag shape. We next exploited the model to explore the diversity of morphotypes that could be generated by varying *dV* and Δ*V* and matched them with existing mutant phenotypes. This approach, focused on the development of the primary inflorescence, allowed us to elaborate on the genetic regulation of the kinetic model of inflorescence development. We propose that the lateral inflorescence meristem fate in tomato is more similar to an immature flower meristem than to the inflorescence meristem of *Arabidopsis*. In the last part of our paper, we extend our thought to spatial regulators that should be integrated in a next step for unraveling the relationships between the different meristems that participate to sympodial growth.

## Introduction

Essentially all cultivated forms of the tomato belong to the species *Solanum lycopersicum*. A large variability exists between cultivars in the form of the plant and leaves, in the number of flowers, or in the shape and color of the fruits. The number and size of the inflorescences are key traits determining potential productivity of the plant and hence understanding the mechanisms that regulate inflorescence architecture is critical.

Historically, inflorescence development in tomato has been studied by a classical forward genetic approach focused on a limited number of mutants, some of which having been found accidentaly in the field and selected for traits that increased yield or facilitated fruit harvest (Emmanuel and Levy, 2002). The different genetic backgrounds in which the mutations had appeared as single alleles and the high plasticity of the flowering process in tomato have impeded research. More recently, tools have been developed for large scale studies in reference genotypes, including generation of mutant populations (Minoia et al., 2010), phenotyping platforms (Ecarnot et al., 2013; Polder et al., 2013) and genome sequencing (Tomato Genome Consortium, 2012), and these progress will undoubtely accelerate functional genomic research. However, carrying out comparative analyses of inflorescence development among different species, either on a gene-by-gene basis or in a modeling attempt, can still be highly constructive today. At the genetic level, the current knowledge obtained in tomato contains sufficient functional and epistasis information that allow to draw a regulatory network of flowering, inspired by what is known in *Arabidopsis*. The first aim of this paper is to give an overview of the knowledge on the subject, based on literature survey. Besides, conceptual frameworks have been recently explored to understand the diversity of inflorescence structures in nature and identify the underlying rules. The second objective of our paper is to exploit these concepts toward modeling the tomato inflorescence, and to test how the model can produce the known mutant phenotypes. This approach allowed us to reciprocally assess the significance of the model and of the genetic network behind.

## Genetic control of inflorescence development

**Figure 1:**
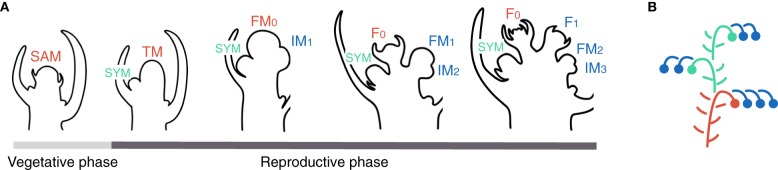
**Inflorescence development and architecture in tomato. (A)** Successive steps of inflorescence development. The vegetative shoot apical meristem (SAM) initiates vegetative phytomers made up of an internode, a leaf and an axillary meristem. When entering floral transition, the SAM takes an intermediate, transitional meristem (TM) fate whereas the last vegetative axillary meristem called the sympodial (SYM) takes over shoot growth. The TM initiates a new phytomer with a prominent inflorescence meristem (IM). TM and IM maturate toward floral meristem (FM) fate and become flowers (F). Each IM initiates another IM in the meantime of maturating to FM. **(B)** Schematic representation of a tomato plant. Colors represent different types of meristems (red: shoot apical meristem, SAM, called transitional meristem, TM, after floral transition; green: sympodial meristem, SYM; blue: inflorescence meristem, IM).

Floral transition of the shoot apical meristem (SAM) is a switch from the production of vegetative phytomers to the initiation of reproductive phytomers. Each vegetative phytomer is made up of an internode, a leaf and an axillary meristem. After the initiation of 6–12 vegetative phytomers forming the initial segment of the plant, the SAM of tomato enters floral transition (Figure 1A) (Sawhney and Greyson, 1972). The last vegetative phytomer is called the sympodial (SYM) because it takes pole position and continues shoot growth after transformation of the SAM into the first inflorescence (Figure 1B). The transitional SAM (transitional meristem, TM), while maturing toward a flower meristem (FM) fate, initiates a new phytomer where, in contrast to vegetative phytomers, the meristematic zone (called inflorescence meristem, IM) is much prominent whereas the subtending leaflike phyllome—or bract—is completely repressed. The IM will reproduce the TM programme, maturing toward the FM fate and initiating a second IM in the meantime. This reiterative process allows endless formation of flowers, providing that maturation and initiation of successive meristems keep in pace.

### Floral transition and flower meristem (FM) fate

There are several excellent reviews on the genetic control of flowering in tomato (Quinet and Kinet, 2007; Samach and Lotan, 2007; Lozano et al., 2009); our focus here will be on functional data updated from the recently published literature and on epistasis studies from which genetic interactions can be inferred. It is worth mentioning first that there is no clear distinction in the literature between regulation of “flowering time” and “inflorescence development” in tomato because mutants that are late- or early- flowering according to the number of vegetative phytomers formed in their initial segment also show abnormalities in their first inflorescence. This is a first indication that the termination of the initial segment by floral transition of the SAM and termination of the lateral branches initiated in the inflorescence obey to the same rules. It must keep in mind, however, that the mutants investigated so far were isolated from modern cultivars, and are the result of strong selection leading to the acquisition of rapid growth cycle and alleviation of environmental requirements for floral transition (Kinet and Peet, 1997) and hence more variability in flowering time might be found in the future by larger exploration of diversity.

**Figure 2:**
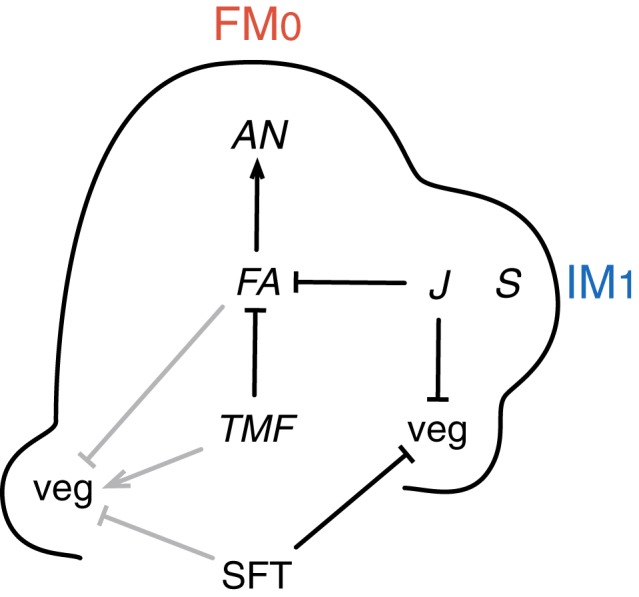
**Genetic control of meristem fate in tomato inflorescence**. The left side of the diagram shows regulatory interactions (gray lines) at floral transition; the right side shows regulatory interactions (black lines) during the development of the inflorescence. Floral transition of the SAM is controlled by upregulation of *FALSIFLORA* (*FA*) in the meristem and by systemic SINGLE FLOWER TRUSS (SFT) signal, which both repress vegetative growth (veg). *TERMINATING FLOWER* (*TMF*) plays an antagonistic role and promotes veg, possibly by repressing *FA*. During inflorescence development, *FA* is required for maturation toward flower meristem (FM) fate, together with activation of the FM identity gene *ANANTHA (AN)*. By contrast, *SFT* is not required for FM identity but represses veg in the lateral inflorescence meristems (IM). This role is shared with *JOINTLESS* (*J*) that represses veg and prevents premature maturation of IM toward FM, possibly by repressing *FA*. By contrast, *COMPOUND INFLORESCENCE* (*S*) accelerates IM maturation. Repression lines and activation arrows do not mean direct interactions.

Interestingly, late-flowering mutants of tomato show an increased propensity to return to vegetative functioning in the inflorescence indicating that common mechanisms are involved in repressing vegetative growth in the SAM and in the lateral meritems initated afterwards in the inflorescence. This is the case of the *falsiflora* (*fa*) and *single flower truss* (*sft*) mutants that produce more vegetative phytomers before floral transition of the SAM, and where leaf production resumes in the inflorescence (Allen and Sussex, 1996; Molinero-Rosales et al., 1999, 2004). On the opposite, overexpression of *FA* or *SFT* accelerates flowering of the initial segment, which produces 3–5 leaves only, and can transform its multi-flowered inflorescence into a single flower (Lifschitz et al., 2006; MacAlister et al., 2012). It can be concluded therefore that *FA* and *SFT* are potent promoters of floral transition in tomato (Figure 2). The late-flowering phenotypes of *fa* and *sft* mutants are additive (Molinero-Rosales et al., 2004; Thouet et al., 2012), indicating that the genes act in parallel pathways.

Only recently, an early flowering mutant of tomato was studied in detail: *terminating flower* (*tmf*) shows the same reduction in vegetative phytomer number in the initial segment and solitary flower phenotypes than plants overexpressing *FA* or *SFT* (MacAlister et al., 2012). Interestingly, *fa* but not *sft* mutation is epistatic to *tmf*, indicating that *TMF* acts upstream of *FA* but independently of *SFT* (Figure 2). Consistently, *FA* is prematurely activated in the *tmf* mutant, although the TM still expresses molecular markers of vegetative meristem fate (MacAlister et al., 2012). The function of *TMF* would thus be to maintain a vegetative SAM.

The promotive role of *FA* and *SFT* genes on floral transition is fully consistent with the fact that they are the tomato orthologs of the *Arabidopsis LEAFY* (*LFY*) and *FLOWERING LOCUS T* (*FT*) genes, respectively (Molinero-Rosales et al., 1999; Lifschitz et al., 2006). *FA*, like *LFY* in *Arabidopsis*, is expressed in the leaf primordia before floral transition, and its expression increases in the meristem at TM stage (Molinero-Rosales et al., 1999; Park et al., 2012; Thouet et al., 2012). *SFT*, like *FT* in *Arabidopsis*, is expressed in the leaves and encodes a systemic florigenic signal (Lifschitz and Eshed, 2006; Lifschitz et al., 2006). The SFT signal is graft transmissible and induces early flowering in tomato and other day-neutral or photoperiodic species (Lifschitz et al., 2006). Floral transition of the SAM thus appears to be regulated in tomato, as in *Arabidopsi*s (Blazquez et al., 1997; Corbesier et al., 2007) by two limiting factors at least: the expression level of *FA* in the SAM and the dosage of systemic SFT (Figure 2). Flowering time, as measured by the number of leaves in the initial segment, is rather stable under various environmental conditions, the major effect being due to the amount of light (Kinet and Peet, 1997) and hence endogeneous clues should be responsible for upregulation of *FA* and *SFT*. An age-dependent increase in expression of *FA* in the SAM was reported (Park et al., 2012) as well as a higher activity of *SFT* in expanded mature leaves than in younger leaves (Shalit et al., 2009). By contrast, *TMF* is expressed predominantly at the periphery of vegetative meristems, extending into initiating vasculature, and decreases slightly at the TM stage (MacAlister et al., 2012). How *TMF*, which encodes a member of the ALOG (Arabidopsis LIGHT-SENSITIVE HYPOCOTYL 1, Oryza G1) family of proteins, might regulate *FA* in the SAM is not known.

*FA* shares with *LFY* the key function of being a FM identity gene. Indeed, the inflorescences of the *fa* mutant are very leafy and made of a combination of vegetative axes with elongated internodes and clumps of indeterminate meristems that are blocked in their development (Allen and Sussex, 1996; Molinero-Rosales et al., 1999). This phenotype is even stronger than that of *lfy* in *Arabidopsis* which shows replacement of flowers by leafy branches but, unlike *fa*, may eventually produce some abnormal flowers (Schultz and Haughn, 1993). A second FM identity gene identified in tomato is *ANANTHA* (*AN*) whose mutation leads to the formation of cauliflower-like masses of meristems where leaves, although still present, are highly suppressed (Allen and Sussex, 1996). The *AN* gene encodes an F-box protein orthologous to UNUSUAL FORMATION OF ORGANS (UFO) which, in *Arabidopsis*, acts as a cofactor of LFY for upregulation of homeotic genes in petal and stamen whorls of the flower (Lee et al., 1997). Thus *AN* and *FA* form a conserved floral specification complex that hallmarks FM fate (Figure 2) (Moyroud et al., 2010). The *fa* mutation is completely epistatic to *an* (Allen and Sussex, 1996) and the expression of *AN* is undetectable in *fa* mutants, indicating that *FA* functions upstream of *AN* (Lippman et al., 2008). Consistently, the expression of *FA* after floral transition of the SAM is higher in maturing FM than in IMs (Thouet et al., 2012) while activation of *AN* occurs later in the FM (Lippman et al., 2008).

### Inflorescence meristem (IM) fate

Contrary to *FA* and *AN*, the loss of *SFT* function does not prevent formation of flowers but hampers continuation of their initiation in the inflorescence: in the *sft* mutant, the reappearance of vegetative axes follows the formation of one or a few normal flowers (Molinero-Rosales et al., 2004; Quinet et al., 2006b), indicating that the *SFT* gene is not required for floral identity but for the maintenance of the floral switch. Consistently, overexpression of *SFT* in different non-allelic flowering mutants caused early termination of the primary segment after 3–4 leaves but did not rescue morphogenetic defects (Shalit et al., 2009). On an *sft* receptor, 35S:SFT donor complemented the inflorescence phenotype as long as the graft was maintained, indicating that permanent emission of SFT signal is required for proper formation of the inflorescence (Lifschitz et al., 2006).

Mutation of the *JOINTLESS* (*J*) gene, like loss of *SFT* function, allows the resumption of vegetative growth in the inflorescence after a few flowers are formed (Szymkowiak and Irish, 1999; Mao et al., 2000; Quinet et al., 2006b), indicating that both genes are required to confer IM identity on meristems that arise after floral transition of the SAM (Figure 2). This is supported by the fact that a very robust one-flower phenotype is obtained by the combination of *sft* with *j* mutation (Thouet et al., 2012). The *J* gene encodes a MADS-box protein of the SHORT VEGETATIVE PHASE (SVP)/AGAMOUS LIKE 24 (AGL24) clade (Mao et al., 2000). *In situ* hybridization and transcriptomic analyses showed that *J* is expressed in the SAM at floral transition (Park et al., 2012) and is later more active in the IMs than in FM (Thouet et al., 2012). Because MADS-box proteins act in complexes, it was previously hypothesized that J interacts with a MADS-box protein induced by systemic SFT protein, and that this complex represses vegetative growth in the newly initiated meristems of the inflorescence (Thouet et al., 2012). Thus, identification of the MADS-box partners of J is important for further functional analyses. Leseberg et al. (2008) found interaction in yeast between J and several other MADS-box proteins of the same sub-families as SUPPRESSOR OF OVEREXPRESSION OF CO1 (SOC1), APETALA1/FRUITFULL (AP1/FUL), and SEPALLATAs (SEPs) in *Arabidopsis*. Functional evidence was obtained for the interaction of J with the MACROCALYX (MC) protein of the AP1/FUL clade, since the expression of an antisense *MC* gene phenocopies the *j* mutation, including the leafy inflorescence phenotype (Vrebalov et al., 2002; Nakano et al., 2012). Interestingly, repression of *MC* also causes conversion of sepals to leaf-like structures, a morphological trait that is also observed in one-flowered *sft* and *j sft* mutants where one of the leafy-sepals is much larger than the others (Molinero-Rosales et al., 2004; Quinet et al., 2006b; Thouet et al., 2012).

### From IM to FM

Gene clusters that are dynamically expressed during meristem maturation have been identified and define a “maturation clock” that can be used to capture the relative maturation state of the meristems in the inflorescence and evaluate their “maturation rate” (Park et al., 2012). This tool offered an explanation to the very early-flowering and single-flower phenotype of the *tmf* mutant, due to premature activation of the FM molecular network, including *FA* and *AN* in the SAM (MacAlister et al., 2012).

In contrast to *tmf*, the *compound inflorescence* (*s*) mutant forms highly branched inflorescences containing tens or hundreds of flowers (Quinet et al., 2006b). At seemingly identical stages, the TM and IM are delayed in maturation in the *s* mutant as compared with WT inflorescences and consequently the time window during which they can initiate a higher order branch is extended (Lippman et al., 2008; Park et al., 2012). This finding indicates that, in WT inflorescence, the *S* gene promotes TM and IM maturation toward the FM fate. The expression pattern of *S* is consistent with this hypothesis, *S* being transiently expressed in the TM and the IM and followed by activation of the FM identity gene *AN* (Figure 2) (Lippman et al., 2008; Park et al., 2012), but the mechanism is not understood. The *S* gene encodes a homeobox domain protein of the WUSCHEL (WUS) family, WOX9, involved in *Arabidopsis* in stem cell maintenance (Wu et al., 2005; Lippman et al., 2008) and hence one possible scenario is that *S* modulates the rate of maturation via the regulation of meristem size. *FA* expression shows little change in expression in the *s* mutant whereas activation of *AN* is much delayed, suggesting that *S* acts downstream of *FA* and upstream of *AN* (Park et al., 2012).

Unexpectedly, the *j* mutation was found to completely override the highly branched phenotype of *s*, indicating that *J* acts antagonistically to *S* and represses early maturation of IM (Thouet et al., 2012). This hypothesis is supported by the fact that in *Arabidopsis*, a MADS-box protein complex that includes the homologs of J, AGL24 and SVP, represses premature activation of FM identity genes (Liu et al., 2009). In this way, J would be essential in the IM to prevent both return to leaf production and premature differentiation (Figure 2). Such a role is consistent with the expression pattern of *J* which is more highly expressed in the IM than in the FM, complementarily to the pattern of the FM identity gene *FA* (Thouet et al., 2012).

## Vegetativeness gain and loss generate a zigzag model

### Model description

Modeling helps to reveal rules underlying repetitive processes such as the construction of the plant body. Two recently proposed models help to comprehend the development of the tomato inflorescence, based on the fact that the arrangement of flowers reflects the spatiotemporal balance between maintenance of meristem indeterminancy and acquisition of floral meristem identity. In their model, Prusinkiewicz et al. (2007) postulate that an inflorescence is built from different meristems that lose their initial “vegetativeness” to become flowers at different times and rates. In the Solanaceae model proposed by Lippman et al. (2008), the branching of the inflorescence depends on the maturation rate of the IM toward FM fate. Both models thus describe meristem development as a continuum—seen alternatively as vegetativeness loss or maturity gain—from initiation to floral commitment.

Our aim here was to construct a simple kinetic model of inflorescence development in tomato. Therefore, we used the term “vegetativeness” after Prusinkiewicz et al. (2007) since it seemed appropriate to describe the frequent resumption of leaf production in the inflorescence, as observed in the *fa*, *sft*, and *j* mutants (see above). In our model, vegetativeness is a complex variable representing the meristem state, with high levels of vegetativeness corresponding to shoot meristem identity and low levels to flower meristem identity. Transcriptomic analyses of individual meristems in tomato allowed to capture gene regulatory networks of different maturation stages and showed that a “molecular clock” drives meristem maturation as a continuous process (Park et al., 2012). We then assumed that vegetativeness could be represented as a continuous function.

**Figure 3:**
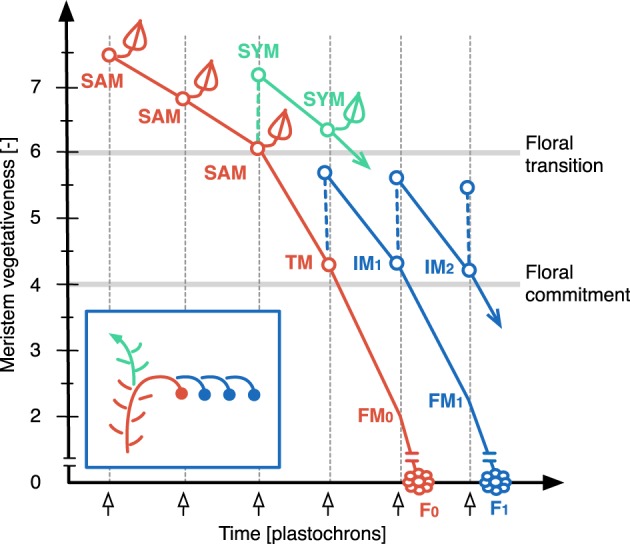
**The zigzag model**. Plots show vegetativeness decline of successive meristems initiated at one-plastochron intervals. Colors represent different types of meristems (red: shoot apical meristem, SAM, called transitional meristem, TM, after floral transition; green: sympodial meristem, SYM; blue: inflorescence meristem, IM). TM and IM maturate toward floral meristem (FM) fate and become flowers (F). See Figure 1 for spatial visualization. Note that the vegetativeness level of the SYM should be higher than shown, since it usually produces more than one leaf.

In Figure 3, the ontogeny of the tomato inflorescence (see Figure 1) is schematized with each line showing the vegetativeness decline of meristems initiated sequentially at one-plastochron intervals. Flowering of cultivated tomato occurs autonomously and hence we assumed that the vegetativeness of the SAM decreases continuously during the vegetative phase of the plant until it passes—at the TM stage—below a permissive threshold for flowering. The last leaf bears an axillary meristem, which is the SYM. Maturation of the TM toward FM fate defines a second phase of vegetativeness decrease during which it initates a lateral IM that will go through the same program: maturate toward FM fate and initiate a lateral meristem. This means that maturation of TM and of successive IMs to FM is slow enough to permit initiation of one IM before they lose indeterminancy. Since IMs are produced iteratively, we postulated that each lateral meristem is initiated at a lower maturity level (i.e., has a higher vegetativeness) than the one from which it was produced. This was expressed by adding Δ*V* to the vegetativeness level of the previous-order meristem at each new meristem initiation event, creating a link between successive meristems. This gave a “zigzag” shape to the meristem dynamics building-up the inflorescence and allowed to simulate the inflorescence of WT plants.

Based on the zigzag dynamics shown in Figure 3, we constructed a mathematical model (Figure S1) in which every meristem was subjected to the same rules:

- Meristem vegetativeness decreases with time following the equation:
  $$V_{i}=V_{i-1}-\frac{dV}{V_{i-1}},$$
  where *V*_*i*_ is the current vegetativeness level of the meristem at plastochrone i, *V*_*i* − 1_ is its vegetativeness one plastochrone before (or at initiation (*V*_0_) see Figure S1) and *dV* is the rate of vegetativeness decrease. We found that a non-linear decrease of the vegetativeness (simply obtained with *dV*/*V*_*i* − 1_) was necessary to stop the production of flowers in WT inflorescences and to account for the vegetative reversions observed in some mutant inflorescences (see below). *dV* can take different values before and after the floral transition. Changes before the transition affects flowering time while changes after the transition have an effect on the architecture of the inflorescence.
- Leaf production (vegetative functioning) is repressed below the floral transition threshold.
- At fixed time points (plastochrons), meristems are allowed to produce a new phytomer, which includes an axillary meristem, unless their vegetativeness is below the floral commitment threshold.
- At initiation, a lateral meristem has a higher vegetativeness level (*V*′_0_), than the meristem that produced the phytomer:
  $$V\prime_{0}=\;V_{p}+\;△V,$$
  where *V*_*p*_ is the vegetativeness (*V*_*i*_) of the previous-order meristem and Δ*V* is the gain of vegetativeness at lateral meristem initiation.

Thus, this simple model is based on two vegetativeness threshold values (floral transition and floral commitment) and two variables: the rate of maturation or vegetativeness decrease (*dV*) and the vegetativeness gain of newly initiated meristems (Δ*V*). In the framework of this study, *dV* was changed after floral transition only, in order to focus on inflorescence development.

**Figure 4:**
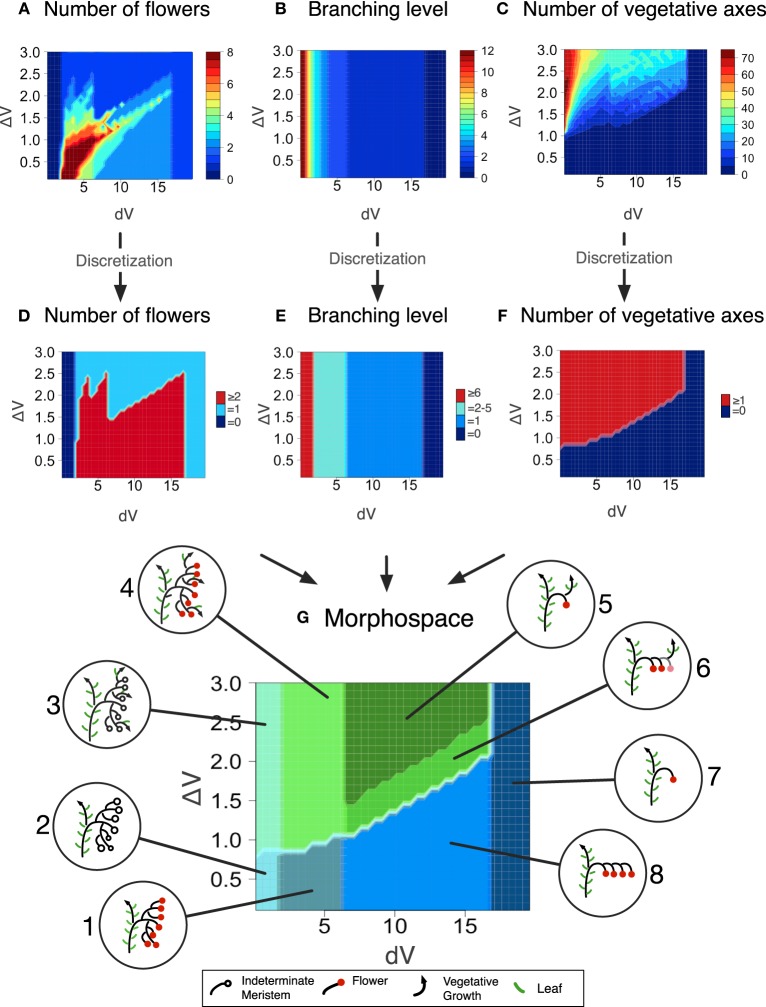
**Morphospace obtained for a range of *dV* and Δ*V* values after floral transition (0 > *dV* > 20 and 0 > Δ*V* > 3)**. Inflorescences generated by the mathematical model were characterized by: **(A)** The number of flowers; **(B)** The branching level; **(C)** The number of vegetative axes. **(D)** Discretization of **(A)** in three classes (0 / 1 / ≥2). **(E)** Discretization of **(B)** in four classes (0 / 1 / 2–5 / ≥6). **(F)** Discretization of **(C)** in two classes (0 / ≥1). **(G)** Superposition of **(D–F)** to form the inflorescence morphospace. Each colored domain corresponds to a morphotype illustrated in inserts. Metrics defining the eight different morphotypes are given in Table 1.

Each output of the model, for any given *dV* and Δ*V* value, is an inflorescence, that was characterized by three metrics describing its topology: the number of flowers before the first occurence of vegetative reversion if any (Figure 4A), the branching level, i.e., the number of phytomers initiated by the TM before being commited to make a flower (Figure 4B) and the number of vegetative axes (Figure 4C). These metrics were arbitrarly discretized, i.e., threshold values were fixed (Table 1) in order to divide the range of morphological variation created by the model (the “morphospace”) in a reduced number of inflorescence types (“morphotypes”) (Figures 4D–G). In order to test whether the model was able to generate known mutant phenotypes, it was run for a range of Δ*V* (from 0 to 3) and *dV* (from 0 to 20) values for a total of 1200 simulations. These ranges were chosen to capture the largest variation of simulation outputs (Figure S2).

**Table 1:** **Combination of metrics describing the first inflorescence topology and used to distinguish eight different morphotypes generated by the model**.

**Morphotype**	**Number of flowers**	**Branching level**	**Number of vegetative axes**
1	≥2	2−5	=0
2	=0	≥6	=0
3	=0	≥6	≥1
4	≥2	2−5	≥1
5	=1	=1	≥1
6	≥2	=1	≥1
7	=1	=0	=0
8	≥2	=1	=0

*Morphotypes are illustrated in Figure 4*.

### The zigzag model generates a morphospace where known mutants find their place

#### Single mutant morphotypes

**Figure 5:**
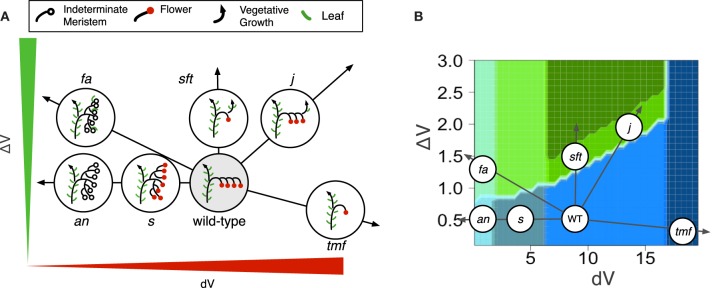
**Phenotypes of known tomato mutants (A) As explained by variations in *dV* and/or Δ*V* and (B) placed in the morphospace of Figure 4**.

In order to test the plausibility of our model, morphotypes were assigned to known mutant phenotypes (Figure S3). Highly branched inflorescences, such as *s, fa* and *an* are found on the left side of the morphospace: they are generated by the model when the vegetativeness of the meristems forming the inflorescence decreases slowly (Figure 5). The slow maturation rate of the IMs allows them to initiate other IMs before being commited to make a flower (Lippman et al., 2008). This hold true for the initial TM as well, so that branched inflorescences always show a proximal fork.

In contrast to *s* mutant, *an* and *fa* never form flowers (Allen and Sussex, 1996; Molinero-Rosales et al., 1999; Quinet et al., 2006b). In our model, the absence of flowers and high branching of the *an* and *fa* inflorescences are explained by an almost null maturation rate of IMs (*dV* value close to zero; Figure 5), reflecting the fact that the meristems never acquire the FM identity. In addition, the return to leaf production in the inflorescence of *fa* mutant would result from an increase in Δ*V* which ultimately causes the vegetativeness level of newly formed lateral meristems to exceed the threshold value for vegetative vs. reproductive programs (floral transition threshold, Figure 3).

On the opposite side of the morphospace created by our model stand mutants with reduced branching and flower numbers, the more extreme one being *tmf* (Figure 5). In our model, its single flower phenotype is obtained by an acceleration of TM maturation (*dV* values > WT), possibly combined with a decrease in Δ*V*. While this increase in *dV* in *tmf* reflects the precocious floral commitment of this mutant, an additional Δ*V* contribution is supported by the vegetative reversions and higher branching observed in *TMF* overexpressors (MacAlister et al., 2012). It is important to emphasize that this interpretation of *tmf* is valid for the SAM of the initial segment only since inflorescences formed later are more or less normal (MacAlister et al., 2012).

Other single-flower mutants of tomato are late flowering and their single-flower phenotype is due to a return to vegetative functioning in the inflorescence, as known for the *sft* mutant (Molinero-Rosales et al., 2004). In our model, the *sft* morphotype is generated by an increase in Δ*V*, which results in the initiation of one or a few flowers before the vegetativeness level of the newly initiated lateral meristem exceeds the threshold value for vegetative functioning. The same phenotype is observed in the inflorescence of the *j* mutant (Szymkowiak and Irish, 1999) but, as explained earlier and unlike *sft*, *j* mutation was shown to accelerate IM maturation (Thouet et al., 2012). That is consistent with our model showing that the morphotype corresponding to *sft* and *j* can be generated by an increase in Δ*V* combined or not with an increase in *dV*.

#### Additivity of dV and ΔV contributions in double mutants

**Figure 6:**
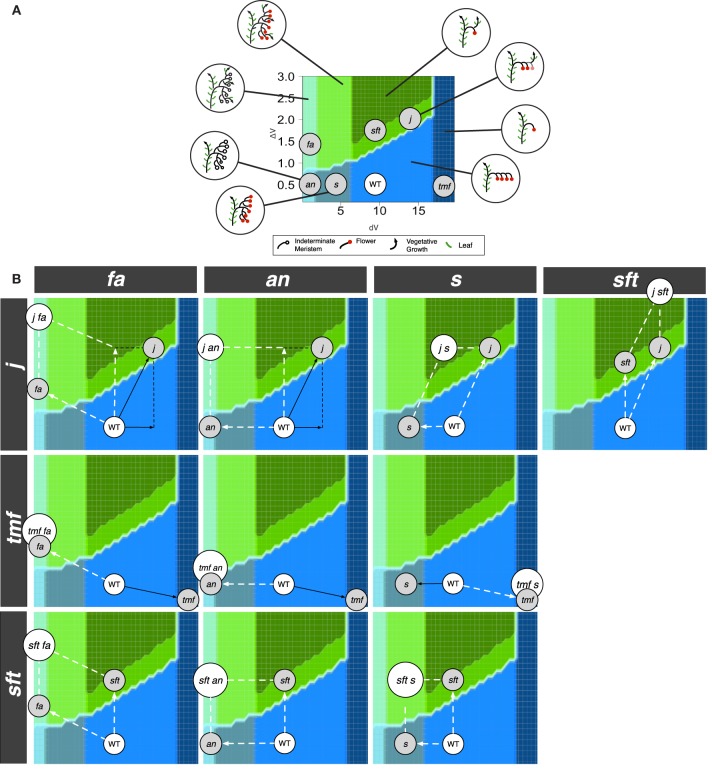
**Positioning of the single and double mutants in the morphospace. (A)** Single mutants. **(B)** Selected double mutants. White arrows represent effective contributions (vectors) of one parent to double mutant phenotypes while black arrows show vectors masked by epistasis.

Double mutant analyses provide additional data to test the consistency of the model and to examine the relative contributions of the two variables *dV* and Δ*V* to deviation from WT inflorescence. For each single mutant, these deviations, or translations, were materialized in the morphospace by vectors (Figure 6A). We therefore evaluated whether double mutant phenotypes could be explained by summing *dV* and/or Δ*V* variations attributed to the single mutations, i.e., if their position in the morphospace (as deduced from their phenotype, Figure S4) could be predicted by the vector resulting from the addition of single mutant vectors. Such an analysis clearly depends on the position attributed to each single mutant in the area of its own morphotype but reciprocally, the phenotype of the double mutants actually provides experimental data to refine the mapping of their parents. Further testing could be performed by combining allelic series, which are not available yet.

We found that, in most cases, our assumption was correct (Figure 6B) since many double mutants fell in the morphotype area pointed by the resultant vector. In these cases, we qualified the interaction between the two genes as “additive.” The most representative example is given by the *s sft* double mutant, combining the high branching of the *s* parent (lower *dV*) and the propensity to return to leaf production of *sft* (higher Δ*V*). This cross produces a morphotype with numerous flowers and vegetative axes that is not found in single mutants (Lippman et al., 2008; Thouet et al., 2012), but was correctly predicted by our model. Additivity was also reported between the flowerless *an* mutant (null *dV*) and *sft*, generating branched flowerless inflorescences with vegetative axes (Lippman et al., 2008) like the *an j* double mutant (Szymkowiak and Irish, 2006). This phenotype belongs to the same morphotype as the *fa* single mutant, supporting that our interpretation was correct. We indeed assumed that the leafy phenotype of *fa* inflorescences was due to the fact that IM are initiated at a higher vegetativeness level, which was translated in our model by a higher Δ*V*. The *fa an* double mutant has the *fa* phenotype, suggesting that *FA* acts upstream of *AN* (Allen and Sussex, 1996), *fa* imposing its Δ*V* contribution over *an* (not illustrated).

Unexpectedly, we also found that the *j s* double mutant phenotype matched the morphotype pointed by the vector resulting of the addition of the two single mutant contributions. Indeed, on the basis of phenotypic analyses, *j* mutation was described as epistatic to the *s* mutation since the inflorescences of the double mutant are indistinguishable from that of the *j* single mutant (Thouet et al., 2012). However, our model shows that this epistatic phenotype could be alternatively explained if we consider that the high *dV* of *j* is able to counterbalance the low *dV* of *s*.

Mutations that alter Δ*V* were also found to be additive, suggesting that “vegetativeness” is a quantitative feature. On the right side of the morphospace, two mutations stimulate a return to vegetative functioning in the inflorescence: *sft* and *j*. Their effect is additive since the double *j sft* mutant returns to vegetative functioning after initiation of a single flower while the single mutants may produce more flowers (Thouet et al., 2012). Moreover, both *sft* and *j* stimulate the development of leaves in *fa* and *an* inflorescences (Szymkowiak and Irish, 2006; Thouet et al., 2012). In *fa* background, the increased Δ*V* due to *sft* or *j* adds on the increment due to *fa* mutation and hence less branches are formed in the double mutants before the vegetativeness of the new meristems exceed the threshold for leaf production (Thouet et al., 2012).

#### Masking effect of extreme dV values

By contrast, mutations that changed *dV* to extreme values, i.e., laying at the extreme left and right sides of the morphospace, did not show additive interactions with *dV* contributions of other mutants: minimum (*fa*, *an*) and maximum (*tmf*) *dV* values were not counterbalanced by intermediate *dV* values of other mutants. This masking effect, herafter qualified as epistatic with respect to the phenotypic traits due to *dV* variations (branching and flower number), can be deduced by the non-coincidence in the morphospace between the double mutant phenotype and the resultant vector obtained from the single mutants.

Epistasis was observed for *fa*, in *fa s* (Thouet et al., 2012), *fa j* (Thouet et al., 2012), and *fa tmf* (MacAlister et al., 2012) double mutants showing highly branched, flowerless inflorescences like *fa*. The *an* mutation was also reported to be epistatic to *s* (Lippman et al., 2008), to *j* (Szymkowiak and Irish, 2006) and to *tmf* (MacAlister et al., 2012) for the same traits. Epistasis of *an* and *fa* finds its biological significance in the fact that *FA* and *AN* genes are indispensable FM identity genes and hence mutations completely block maturation to FM fate.

On the right side of the morphospace, the high *dV tmf* mutant is epistatic to *s* with respect to first inflorescence architecture (MacAlister et al., 2012). Epistasis of *tmf* over *s* mutation is due to the early activation of *FA* and *AN* (MacAlister et al., 2012), forcing maturation and preventing any branching of the inflorescence. The double *j tmf* mutant has not been described so far but can be predicted to have *tmf* phenotype as well.

## Integrating genes within the zigzag model

In *Arabidopsis*, modeling of inflorescence architecture focused on two genes considered as master regulators: *LFY*, which reduces vegetativeness in meristems and *TERMINAL FLOWER 1* (*TFL1*) which increases vegetativeness (Prusinkiewicz et al., 2007). The “transient model” proposed by Prusinkiewicz et al. postulated that lateral meristems are initiated at a transient state of vegetativeness and therafter become a flower or revert to produce a branch. It yielded different types of inflorescence according to the relative time length different meristems take to achieve flowering and it was therefore used to address the adaptative and evolutionary value of inflorescence architectures. This model was deeply discussed because alternative rules could yield the same observed types of inflorescences and because the reduction of a complex developmental process to a pair of antagonistic genes seemed oversimplified (Alvarez-Buylla et al., 2007; Winther, 2012). However, although the transient model excluded some special cases of inflorescence architecture (Prenner et al., 2009), its unifying goal has been largely acknowledged (e.g., Castel et al., 2010). More recently, another modeling approach was used considering groups of genes or “hubs” that contribute to the function of key regulators of floral transition in *Arabidopsis* (Jaeger et al., 2013). This approach allowed to generate the racemose inflorescence, providing that the *TFL1* hub is upregulated in proportion of the floral inductive signal FT. Feedback loops then establish a stable state with *TFL1* repressing flowering and maintaining indeterminancy at the center of the SAM (called IM) whereas *LFY* is expressed and flowers are initiated on the flanks of the meristem.

Our tomato model uses the same terminology of vegetativeness as Prusinkiewicz et al. (2007) and describes meristem maturation as a continuous decrease of vegetativeness (*dV*) but the overall dynamics are different. While Prusienkewicz et al. introduced a transient state in lateral meristem fate, a key feature of our model is that the maturation state of a lateral meristem depends on the meristem from which it derives. This link is expressed by the variable Δ*V* and might be established in the meristems by the diffusive properties of some regulators, as postulated by other authors (Alvarez-Buylla et al., 2007).

At the genetic level, our model does not incorporate *TFL1* because, as observed in other species forming cymose inflorescences, the tomato homolog of *TFL1* is not expressed in the SAM at floral transition or during inflorescence development (Thouet et al., 2008; see below). However, the mapping of known inflorescence mutants into the morphospace created by the model (Figures 5, 6) allowed us to inferre the contribution of the corresponding genes in the regulation of *dV* and/or Δ*V*, summarized in Table 2. The emerging view is undoubtedly simplified since the activity of each gene is likely to reflect system-level changes in planta but it incorporates, without a priori assumption, all genes affecting inflorescence architecture that have been characterized so far in tomato.

**Table 2:** **Contribution of genes to regulation of the two variables used for modeling the tomato inflorescence, as inferred from the position of the loss-of-function mutants in the morphospace**.

**Gene**	**Vegetativeness gain of newly initiated meristems (Δ*V*)**	**Rate of vegetativeness decrease (*dV*)**
*AN*		+
*FA*	−	+
*S*		+
*SFT*	−	
*J*	−	−
*TMF*	+	−

*(−) means that the gene activity has a negative impact on the parameter; (+) means that the gene activity has a positive impact on the parameter; lack of sign means that no correlation was found*.

*TMF* is the only gene in Table 2 that increases vegetativeness and this was shown by MacAlister et al. (2012) to occur by repression of a subset of genes regulating floral commitment, including *FA*. The *TMF* gene could thus play in tomato the role of *TFL1* repressing *LFY* in *Arabidopsis* but this role would be limited to the SAM (MacAlister et al., 2012). Floral transition is marked by the upregulation of *FA* in meristem, but unlike *LFY* in *Arabidopsis*, the activation of *FA* is not limited to subdomains (Thouet et al., 2012) and terminates vegetative growth. Thus, repression of *FA* by *TMF* is temporal and not spatial, and the role of *TMF* is to maintain the vegetative fate and not “just” indeterminancy, unlike *TFL1* in *Arabidopsis*.

The ontogeny of the inflorescence in tomato proceeds by iterative initation of new lateral meristems and the vegetativeness in these meristems is lowered by *FA*, *J*, and *SFT* (Table 2): if any of these genes is not functional, the tomato inflorescence contains leaves (Molinero-Rosales et al., 1999, 2004; Szymkowiak and Irish, 2006). Their lowered vegetativeness justifies that lateral meristems in the inflorescence are called IM, since they are intermediate between the two categories of meristems: the vegetative meristem which primarily produces leaves and stems and the FM which produces only floral organs (Prenner et al., 2009). Importantly, IM fate determines in tomato the time window during which meristems have the ability to branch and hence there is a close relationship between the duration of the IM fate and the number of branches in the inflorescence (Lippman et al., 2008) as shown by the large impact of varying *dV* on the morphotype. We discussed above the limitation of our model for expressing epistasis relationships (extreme *dV* values) and hence we will point here the functions of two genes: *S*, which accelerates the transition from IM to FM fate and *J*, which has the opposite effect (Table 2). The function of genes such as *J* is critical to built multiflowered inflorescences since premature achievement of FM fate would lead to termination of the inflorescence. This function of *J* in the IM was suggested to proceed through negative feedback from *J* to *FA* (Thouet et al., 2012) and must be transient as maturation proceeds toward FM fate. Thus a J/FA balance might have a pivotal role in the regulation of inflorescence development in tomato. How the flowering signal SFT regulates this balancing remains to be clarified but the facts that it promotes floral transition independently of *FA* (Molinero-Rosales et al., 2004) and interacts with *J* which refrains flower development (Thouet et al., 2012) establish clear parallels with the mechanism of interlocking loops disclosed by Jaeger et al. (2013).

Interestingly, the homologs of *J* in *Arabidopsis*, the MADS box genes *AGL24* and *SVP*, are involved in repressing differentiation at the early stages of FM formation and are therefore, together with FM identity genes, parts of regulatory loops timing meristem maturation (Liu et al., 2009; Wagner, 2009). It is worth emphasizing the importance of the “rate of maturation” (our *dV* variable) in this step. We then propose that the IM in tomato behaves as an immature FM in *Arabidopsis* whereas the IM in *Arabidopsis* (regulated by *TFL1*) is more similar to a vegetative meristem in tomato. This hypothesis could be tested by searching for conserved genes, interactions and dynamics within the gene regulatory networks of these meristems; we believe that this approach could provide novel insights into the understanding of inflorescence architectures.

## Perspectives from a side view

Our reasoning has so far been focused on the temporal regulation of meristem fate by a developmental programme, but spatial regulation is intricately linked to the timing. At floral transition indeed, three meristems of a different fate are adjacent to each other: the vegetative SYM, the TM and the first IM (Figure 1A).

Importantly, the SYM does not enter floral transition at the same time as the SAM but will first initiate 3 vegetative phytomers before forming an inflorescence itself; the growth of the plant will then be continued by a second order sympodial segment and so on, indefinitely. This regular iteration of 3-leaf sympodial segments is regulated by the *SELF PRUNING* gene, the closest homolog of *TERMINAL FLOWER 1* in *Arabidopsis* (Pnueli et al., 1998), which is expressed in the SYM and other vegetative axillary meristems but not in the SAM at floral transition (Thouet et al., 2008). Consistently, *sp* mutation does not affect floral transition of the initial segment and does not have any impact on inflorescence architecture (Pnueli et al., 1998). In *sp* mutant, termination of successive sympodial segments occurs with less leaves and ends with two consecutive inflorescences, leading to a determinate growth. This trait facilitates mechanical harvesting of the fruits and hence the *sp* mutation was introduced for breeding “determinate” cultivars used in tomato industry. This phenotype however depends on *SFT* dosage: in *sft*/+ heterozygote background, early termination of sympodial units due to *sp* mutation is overcome (Jiang et al., 2013) and in *sft*/*sft* homozygote background, sympodial growth is suppressed as in *sft* single mutant (Molinero-Rosales et al., 2004). The effect of *sft*/+ heterozygosity in *sp* cultivars leads to a dramatic increase in inflorescence number per plant and thereby in yield (Krieger et al., 2010). By contrast to the fact that the SYM is more sensitive than the SAM to *SP* inactivation, the opposite is observed in the differential response to *SFT*: flowering of plants overexpressing *SFT* is indeed much accelerated in the initial segment but the sympodial segments still initiate 2 or 3 leaves (Lifschitz et al., 2006; Shalit et al., 2009). It was therefore concluded that the SP/SFT balance regulates shoot architecture and sympodial development in tomato (Shalit et al., 2009). It is interesting to note that the *tmf* mutation, like 35S:SFT, affects only the initial segment of the plant (MacAlister et al., 2012) suggesting that *TMF* is also checked by *SP* in the sympodial segments.

Although WT inflorescence architecture is not affected by *sp* mutation, overexpression of *SP* results in the replacement of flowers by leaves (Pnueli et al., 1998), indicating that ectopic expression of *SP* in the inflorescence promotes vegetative functioning. Consistently, vegetative meristems that arise in mutant inflorescences returning to leaf initiation after formation of normal flowers share regulatory features with the SYM as shown for *j* (Szymkowiak and Irish, 2006) and express *SP* as shown for *sft* (Thouet et al., 2008). Consequently, these vegetative axes can usurp the pole position to the canonical SYM forming a “pseudo-shoot” that continuates the initial segment. This occurs when the inflorescence forms a single flower before the vegetative axis is initiated, as observed in strong *sft* mutant (Molinero-Rosales et al., 2004), otherwise the SYM remains dominant. Variability in the number of flowers in the inflorescence of *sft* may be due to allele strength, but also to the influence of the environment since the one-flower phenotype is more frequent in winter than in summer (Quinet et al., 2006b; Park et al., 2012). Interestingly, the environmental conditions that reveal the plasticity of the *sft* phenotype, light quantity and quality, are also those that are known to influence the correlative influence and dominance relationships between lateral meristems.

Another mutant where pseudo-shoots originating from the inflorescence were described is *uniflora* (*uf*) (Lifschitz et al., 2006). In this late flowering mutant, however, no lateral meristem is formed after conversion of the SAM into a flower (Dielen et al., 1998) and hence the origin of the meristem that continues the primary shoot is not clear. The incapacity of *uf* mutant to initiate lateral meristems in the inflorescence explains that the solitary flower phenotype is epistatic to mutations that affect IM fate, such as *s* and *j* (Quinet et al., 2006a). However, *uf* also shows a strong light-dose dependent flowering: the mutant is much delayed when the light integral is low (Dielen et al., 2004). Interestingly, when *uf* plants are transferred from favorable to unfavorable conditions, the number of leaves below the first flower does not show a continuous but a step increase, as if a sympodial segment was recruted in the main axis. Consistent with this hypothesis, *sp* mutation partially compensates late-flowering of *uf* (Quinet et al., 2006a). We therefore hypothesize that *uf* mutation causes a general defect in lateral meristem initiation and/or development rather than affecting flowering *per se*. Other mutants indeed illustrate the basic link between plant branching and inflorescence development in tomato. For example the *blind* (*bl*) mutant fails to initate axillary meristems, including the SYM, so that sympodial growth is completely suppressed, and shows dramatic reduction in inflorescence branching (Schmitz et al., 2002). Most interestingly, the *bl* inflorescence consists of one to a few flowers that tend to be fused and fasciated suggesting incomplete separation of meristems. The *Bl* gene encodes a Myb transcription factor and is expressed in prospective and actual boundaries separating lateral meristems from the SAM (Schmitz et al., 2002; Busch et al., 2011). This pattern emphasizes the importance of proper separation of adjacent meristems for specification of different fates: in *bl* mutant, the FM fate obviously “invades” the lateral IM so that siamese flowers are formed.

At later stages, a separation remains between flowers and the rest of the inflorescence in tomato: the abscission zone. The jointless pedicel character gave its name to the *j* mutants but this was considered as a side effect of the mutation since expression of *J* was not detected in the flower pedicel (Szymkowiak and Irish, 2006). Only recently were contradictory patterns published, showing expression of *J* in the pedicel primordium at early stages of flower development (Liu et al., 2014). Amazingly, trancriptomic analyses showed that branching genes such as *Bl*, boundary genes such as *Goblet* (*Gob*) that is homologous to the *Arabidopsis CUP SHAPED COTYLEDON* genes (Berger et al., 2009) and meristematic genes of the *WUS* family contribute to the development of the abscission region, which supports the idea that the cells of the abscission zone are arrested meristematic cells (Nakano et al., 2013). Interestingly, members of this genetic network are also involved in regulating compound leaf development (Blein et al., 2008; Busch et al., 2011). These findings provide novel insight into the pleiotropic effects of flowering genes on abscission zone development and leaf morphology (Shalit et al., 2009) and suggest that in tomato, partition of adjacent meristems during inflorescence formation, disjunction of flowers at a later stage of development and leaflet formation are cell separation processes sharing common regulatory pathways.

## Conclusion

We presented here a first attempt to link tomato flowering genes into a coherent network. Such network was supported by a mathematical model that was able to generate the phenotypes of a large range of single and double inflorescence mutants. The model is based on the maturation kinetics of the successive meristems that elaborate the inflorescence and create a zigzag dynamics. Spatially, the formation of the inflorescence requires territorialization of adjacent meristematic domains to allow separation of meristem identities. This seems to involve in tomato conserved mechanisms regulating cell separation processes such as axillary meristem initiation, abscission zone development and leaflet formation. A challenging question for the future will be to integrate the spatial dynamics into the temporal models of inflorescence development, and to identify the signaling molecules that orchestrate the morphogenetic plan.

### Conflict of interest statement

The authors declare that the research was conducted in the absence of any commercial or financial relationships that could be construed as a potential conflict of interest.
