# Supplementary material for: Inflorescence development in tomato: gene functions within a zigzag model
Source: Front Plant Sci. 2014 Mar 31;5:121. doi: 10.3389/fpls.2014.00121 (PMC3978268; doi:10.3389/fpls.2014.00121)
Supplement: Supplementary file 1 [file DataSheet1.PDF]

Supplementary Material

Inflorescence development in tomato : gene functions within a zigzag model

Périlleux C*§, Lobet G§, Tocquin P

Laboratory of Plant Physiology, PhytoSYSTEMS, Department of Life Sciences, University of Liège, Liège, Belgium
* **Correspondence:** Claire Périlleux, Laboratory of Plant Physiology, PhytoSYSTEMS, Department of Life Sciences, University of Liège, Boulevard du Rectorat 27, 4000, Liège, Belgium
cperilleux@ulg.ac.be

§ both authors contributed equally to this work

1. Supplementary Figures

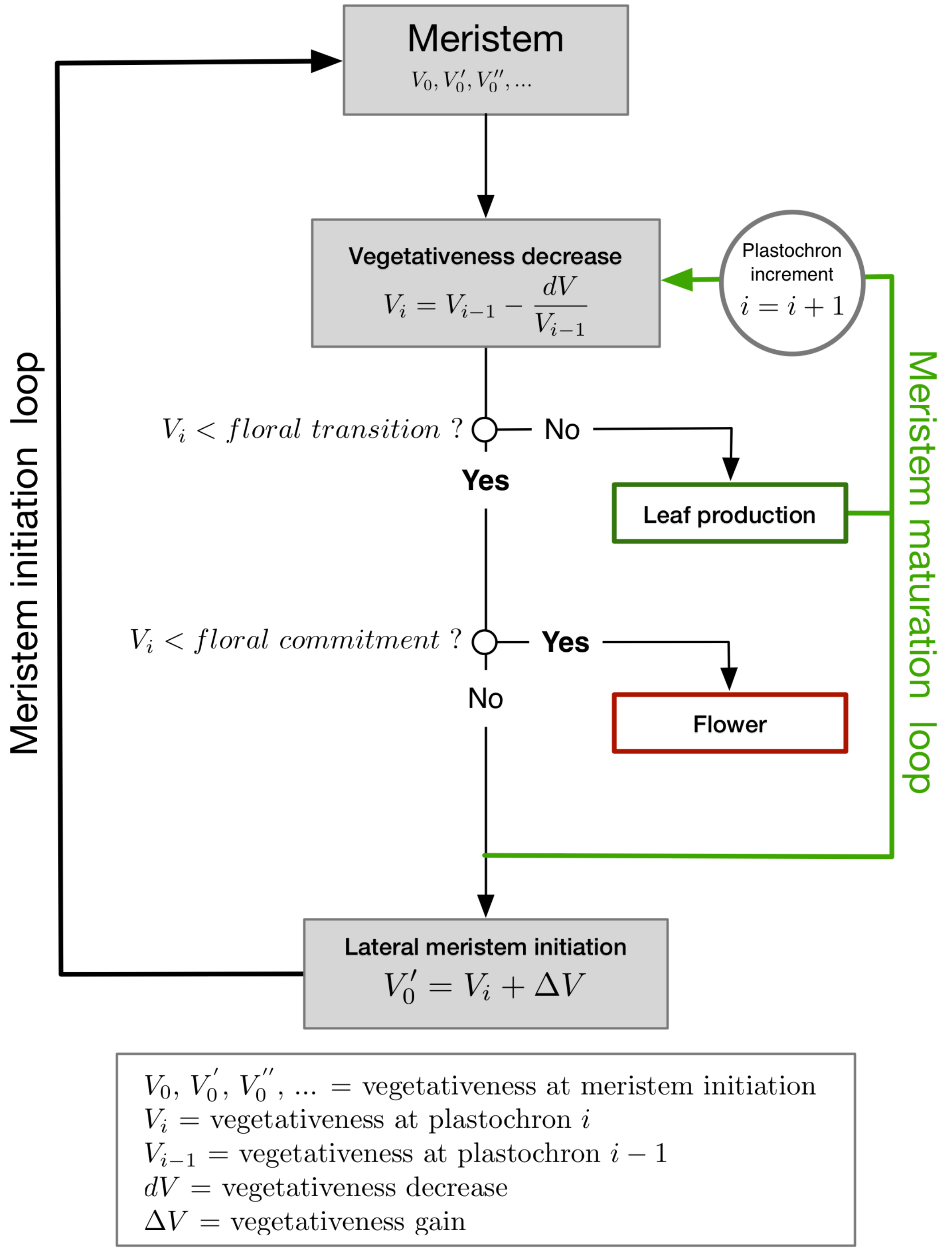

Supplementary Figure 1. Schematic representation of the 'zigzag' model.

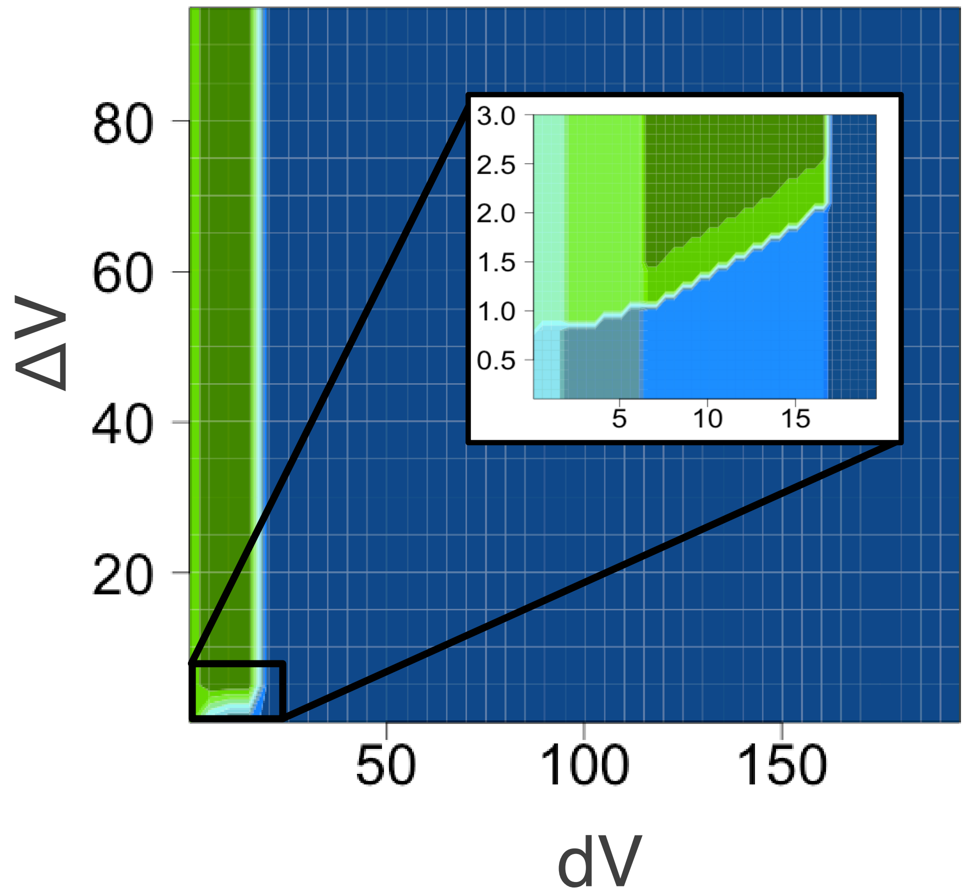

Supplementary Figure 2: Morphospace generated with a wide range of dV and ΔV values. The insert shows the domain used for mapping known mutants.

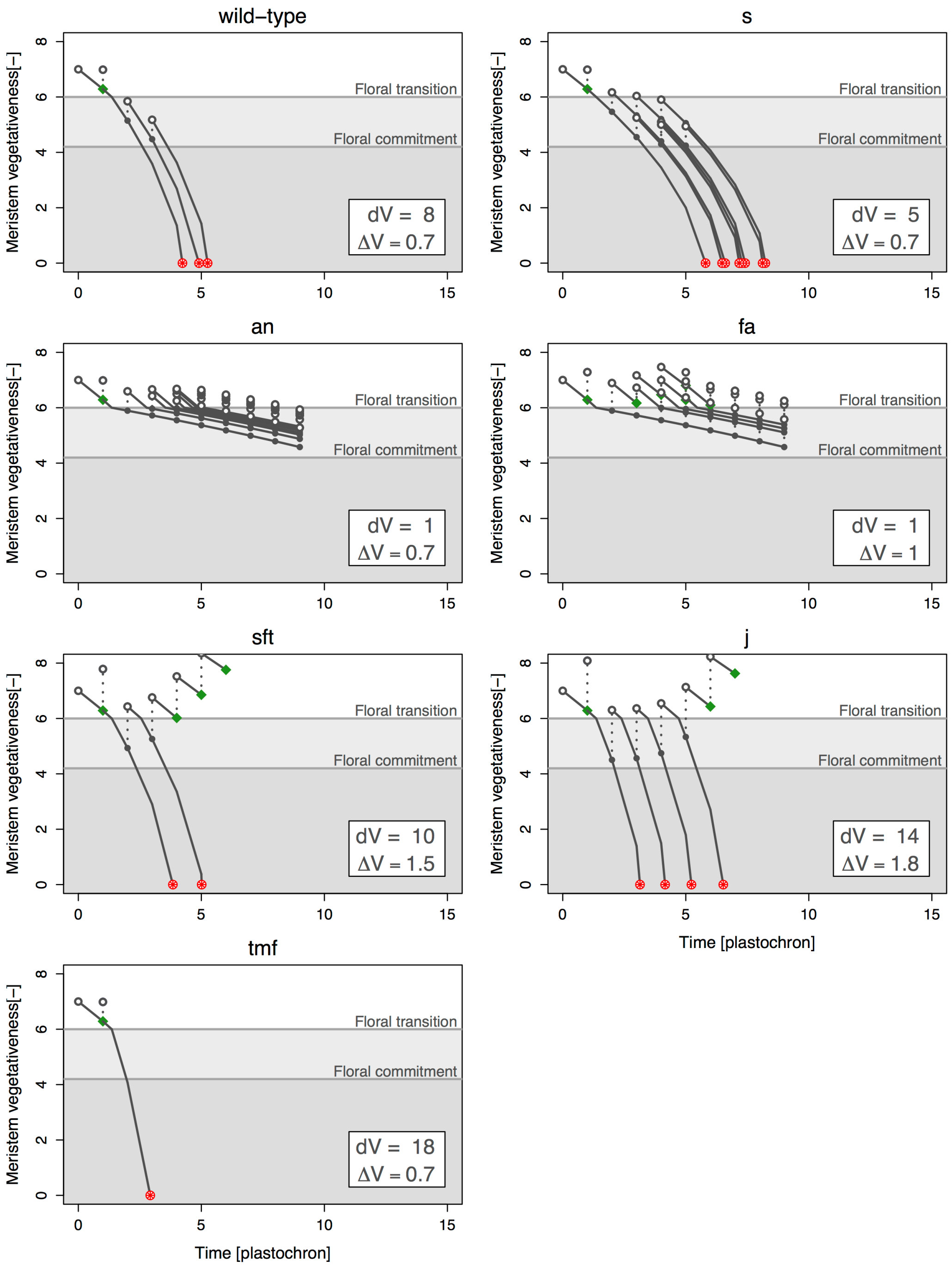

Supplementary Figure 3. Simulation output for the different single mutants. Parameters (dV and ΔV) were chosen according to figure 5. Each meristem is represented by one plot. Initiation of axillary meristems is represented by an open circle, which is connected by a dotted line (ΔV) to closed circles on parent meristem lines. Green diamonds represent leaves. For the sake of clarity, the development of sympodial and other vegetative axes was stopped after one plastochron. Red wheels represent flowers.

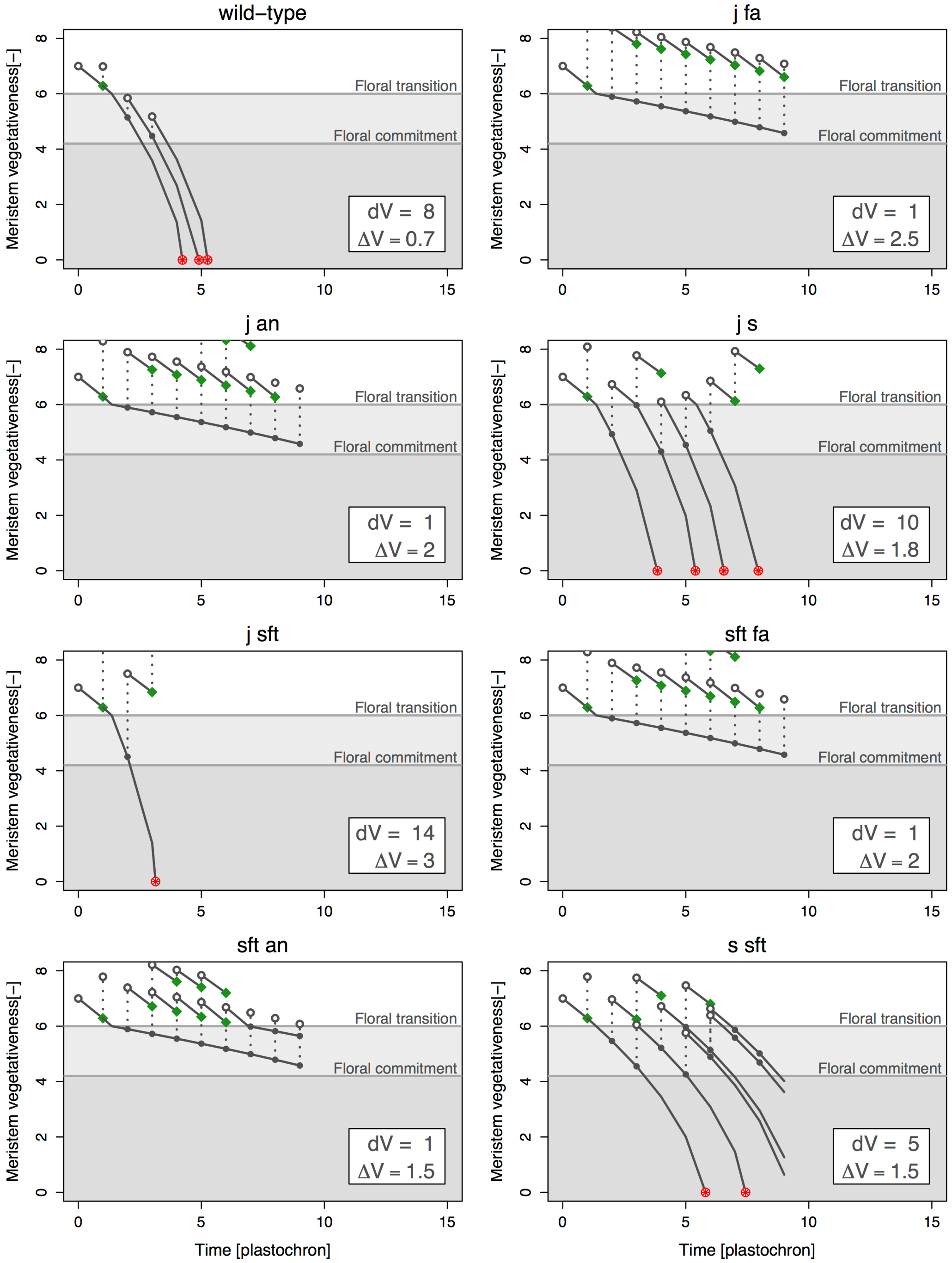

Supplementary Figure 4. Simulation output for the different double mutants. Parameters (dV and ΔV) were chosen according to Figure 6. Each meristem is represented by one plot. Initiation of axillary meristems is represented by an open circle, which is connected by a dotted line (ΔV) to closed circles on parent meristem lines. Green diamonds represent leaves. For the sake of clarity, the development of sympodial and other vegetative axes was stopped after one plastochron. Red wheels represent flowers.
